# The role of product art design based on a fuzzy decision support system in improving user interaction experience

**DOI:** 10.1371/journal.pone.0321477

**Published:** 2025-05-22

**Authors:** Yuqiao Liu, Shuai Zhang

**Affiliations:** Art and Design College, Shenyang Ligong University, Shenyang, China; Industrial University of Ho Chi Minh City, VIET NAM

## Abstract

User interaction for product selection relies on its design and technical support to improve the quality of the experience. Decision support systems are incorporated to leverage user experience through product interactions. This article introduces an interaction-based fuzzy decision support (FDS) system to meet user demands in product design through suggestions for user interaction. The proposed system models the maximum possible interaction features through previous user experiences and reviews. Based on these two factors, the fuzzy decisions for interaction improvement or product design modification are identified through likelihood. This likelihood is a variant between lower and higher fuzzy combinations for maximum interaction pursued by the user. The fuzzy process develops multiple higher-order recommendation variants from the interaction computed to improve the user experience. The lower-order variants recommend different product design features to increase the interaction rate. Thus, the decision process determines the need for adaptability through interactive platforms to achieve a better experience. This methodology aimed to improve the interaction rate of 97.4% with better impacts on product design and modification using likelihood variants. The user experience assessment is performed using the higher-order variants with a better user adaptability rate of 98.9%, maximizing the recommendations.

## 1. Introduction and related works

Decision support system (DSS) technology is used in product design. DSS provides effective decision-making services to select products based on designs and capabilities [1]. DSS reduces the computational complexity while performing tasks for the users. An improved DSS-based model is used for product designs in manufacturing systems [2]. The DSS model helps users select products according to their designs, demands, features, and quality. The DSS model also illustrates the actual capability of the products to the users [3]. The model reduces the computational cost and latency ratio of the process. An enhanced DSS selection approach is also used for product design [4]. The exact expectation features are analyzed via DSS for the product design. The features contain optimal information for the product design that eliminates energy consumption in decision-making processes. The DSS approach enhances the systems’ overall performance and feasibility range [5,6].

Improving user interaction is a crucial task to perform in product designing systems. User interaction services are improved based on expectations gathered from feedback [7]. A feedback-based user interaction improvement method is used for product designs. The feedback is collected from users via certain questionnaires [8]. The feedback produces an effective dataset containing users’ expectations of products and interactions. The method analyzes the features that improve user interaction services [9]. User interaction services are a must to improve the overall product quality level of the products during design phases [10]. An axiomatic theory-based user interaction improvement approach is also used in product design. The axiomatic theory evaluates the interaction services as necessary. The evaluated data is used to enhance the conceptual designing phases of the product in manufacturing systems [11,12].

Optimization methods are used to eliminate the problems that cause damage to the product design process [13]. Optimization methods are widely used to enlarge the efficiency and feasibility range of the designing process. A customer optimization route and evaluation (CORE) method is used for product design [14]. The CORE method is used to identify the exact cause of optimization problems during product design. The CORE method analyzes the market’s needs and designs via feedback [15]. The feedback collected from users contains features and factors for product designs. The CORE method enhances the performance level of the product designs [16]. A back propagation neural network (BPNN) algorithm-based optimization model is used for product design. The BPNN algorithm uses a feature extraction technique that extracts the features from the dataset. The BPNN algorithm reduces the optimization problems, improving the product quality range for the users [17,18].

In real-world applications, user replies may frequently be subjective, conflicting, or ambiguous, which might impact current methods of product design that depend on clear, direct input. In an attempt to meet the demands of a diverse customer base, traditional product design processes ignore these nuances and don’t consider user preferences. Design decisions could be too inflexible or inadequately responsive to user desires due to existing methodologies’ difficulties to analyze this ambiguous input in real time. The Interaction-Based Fuzzy Decision Support System (IB-FDSS) is proposed in this research to fill this need; it uses fuzzy logic to understand and handle contradictory and unclear user comments on product artwork. More adaptable, flexible, and accurate design choices are made possible by IB-FDSS by translating imprecise user inputs into degrees of confidence. In addition to making the design process more efficient, this method ensures that the end product lives up to consumer expectations. Fuzzy logic is a game-changer in product art design because it can manage unclear input and improve user interaction experiences even with loud or confusing data.

The following description provides a detailed explanation of previous works from different authors related to the proposed concept. The consecutive section follows the proposed method discussion and description using fuzzy optimization and order derivatives to improve user experience. The accounted data is utilized later in the article to analyze the proposed method’s performance. The summary and references of the article are presented in the final part of the article.

Yin et al. [19] developed a new evaluation method for product-service systems. The approach suggests that the analytic network process and niche theory should be used to appraise product service system schemes. It calculates the importance of customer value and compares the advantages of different value delivery schemes. The ranking order is decided according to the value delivered to the customer, and the result was validated on CNC machine tool service systems. Zhou et al. [20] proposed an FMEA-based method to analyze product design risk. The technique provides a new way for risk analysis using PFMEA under uncertainty. It constructs grey causality and interaction vectors to describe causal relationships and interactions among failure modes. The method considers cost and time uncertainties to prioritize failure modes and identify optimal design solutions. Liu et al. [21] developed an agent-based model for new product development user engagement. The method models the engagement of users in product development using agent-based simulation. The model combines the two engagement processes, direct and indirect, through which users share knowledge and interact with the product. The findings show nonlinear user engagement growth influenced by social networks and past experiences.

Arnemann et al. [22] designed a gamification-based system that monitored resource consumption. The technique traces resource use by-products and machinery to estimate the carbon footprint. It combines sensor technology with applications working across platforms to realize integration among data and gamification methods. The application provides an intuitive interface for real-time environmental impacts and changes in the production process. Liu et al. [23] contributed a spherical fuzzy bipartite graph-based methodology for assistive product design. The methodology provides a scheme for the prioritization of design requirements based on the needs expressed by patients. The method represents the internal relationships in the Product Requirement-Design Requirement matrix with the help of a spherical fuzzy bipartite graph. The verification is done by the design of assistive products for pneumoconiosis patients. Sun et al. [24] proposed a decision-making approach that could assess product designs by an improved TOPSIS and GRP method. The approach integrated picture fuzzy sets with the multi-criteria decision-making process. It made criteria choices and weight determination through picture fuzzy numbers and entropy. The revealed method applies TOPSIS and weighted Mahalanobis distance to display an optimal design alternative.

Hu et al. [25] developed a product optimization design model driven by user needs. The method ranked user requirements with the Kano Model and Pairwise Analysis. The method transforms user needs into technical solutions using FAST and Quality Function Deployment theories. The method amalgamates the solutions and applies a game theory model for optimum design, improving user satisfaction. Luo et al. [26] presented a multi-criteria decision-making method for manufacturing resource allocation. The proposed method suggests a model to evaluate the allocation of resources under complex product systems. It uses intuitionistic fuzzy sets and hybrid fuzzy methods to deal with the uncertainties in the evaluation criteria. The model adopts the hybrid IFIE-TOPSIS for ranking and finding the best allocation scheme [27]. Singer et al. [28] developed an interval-valued intuitionistic fuzzy analytic hierarchy process model. The methodology identifies key criteria for purchasing non-wood forest products using interval-valued intuitionistic fuzzy AHP. It evaluates six main and thirty sub-criteria to determine the importance of these criteria. The method finds health aspects as the most critical criterion, where the health benefits, price of the products, and nutritional value remain the key sub-criteria.

Ma et al. [29] developed an integrated design concept evaluation model using interval-valued picture fuzzy sets and improved GRP. The proposed method combines models to build a set for evaluating design concepts. It builds weights for the criteria and decision-makers through fuzzy sets and grey relational projection. The best design concepts should be identified to enhance the product development process. Yu et al. [30] provided a scheme for evaluating interactive waiting experiences of mobile internet products using machine learning. The approach analyzes factors that cause negative user emotions by waiting time. The method uses machine learning in cost assessment for an interactive waiting experience. The method proposes design strategies to enhance the user experience and increase product usage. Yang et al. [31] developed the Pythagorean fuzzy Bonferroni mean with a weighted interaction operator. The proposed approach is based on constructing a decision-making tool using Pythagorean fuzzy Bonferroni means with a weighted interaction operator in the combination of online ratings. One of the major limitations tackled in previous techniques is the information aggregation of fuzzy sets and expert knowledge. The proposed approach is tested using passenger car rankings.

Borsci et al. [32] designed a chatbot usability scale for interaction with AI-based conversational agents. Based on the literature review and user testing, the technique comes up with quality attributes for chatbots. The method develops BOT-Check, a checklist and BUS-15, a 15-item satisfaction questionnaire. The method validates these tools for chatbot interaction and finds good reliability with potential for broader application. Classi et al. [33] presented a model of redesign decisions for large-scale complex systems. The methodology is applied to refresh planning and presents fuzzy multi-attribute decision-making techniques to technology choices. The technique appraises the competing options in the technologies selected using a systems thinking approach for better decision-making. The approach fills major gaps in the existing literature by considering multiple factors and redesigning drivers. Chen et al. [34] contributed an interactive design framework of children’s apps for enhanced emotional experiences. The approach develops a framework to evoke three positive emotions in children through educational apps. The emotional design scale analyzes 72 apps from Australian and Chinese App Stores. The approach indicates that focusing on emotional design improves user ratings and offers better emotional benefits for parent-child interactions. Momena et al. [35] developed a solution strategy for the sustainable design of additive manufacturing. The proposed method develops an aggregation approach to combine the decision-maker’s data into Pythagorean fuzzy numbers. It combines the approach with the Pythagorean fuzzy TOPSIS approach to develop a hybrid multi-criteria group decision-making methodology. The approach is applied to the material selection process in additive manufacturing and effectively represents knowledge.

Sitek et al. [36] suggested the decision support model for handling customer orders in the business chain. Since the epidemic and its restrictions and limits on commerce and services have been in effect for some time, this technique of placing and completing orders has become more prevalent. The author has shown two possible implementations of the concept. A proprietary technique incorporating evolutionary processes (e.g., specific representations, repair mechanisms, genetic operators, etc.) uses constraint logic programming and dedicated heuristics, whereas the first relies on mathematical modelling and programming. We have also established protocols for addressing constraints and solving them.

Vahidnia et al. [37] proposed an ontology-based web decision support system to find entertainment points of interest in an urban area. The goal is to make recommendations tailored to the user’s location, age, activity type, and other parameters. As a knowledge base in the Protégé environment, this model has created a domain for entertainment centres using Web Ontology Language (OWL), which allows for managing visitor requests and concerns. Based on a client-server architecture, the web-based DSS uses tools like Flask and Werkzeug. So, it’s feasible to use HermiT-based ontology reasoning to find the correct hub and then do a semantic search on associated classes.

Objectives of the study

- Developing a fuzzy decision support system (FDS) that evaluates and optimizes product art design elements, such as usability, aesthetics, and functionality, to improve user engagement and satisfaction.
- Integrating fuzzy logic to analyze subjective user feedback and objective performance metrics enables designers to make informed decisions on improving product art design.
- Exploring the role of advanced computational techniques, such as fuzzy systems, in harmonizing artistic creativity and technical functionality, fostering innovative design solutions that resonate with diverse user preferences.

## 2. Data description

**Fig 1:**
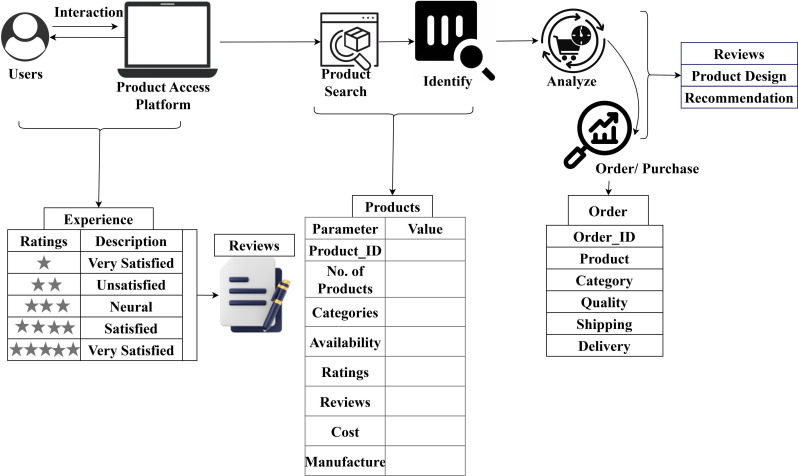
Roadmap and data descriptions of interactive system.

This interaction-based fuzzy DSS for product design assessment integrates three data sources (10.1038/s41597-023-02741-8; 10.3390/informatics9040085). The user experience is computed using reviews and recommendations for various product purchases in an e-commerce platform. Considering the role of users, the interaction efficiency is identified based on products and their likelihood. The likelihood is split into the high and low variants by computing $\;\frac{maximum\;reviews}{users}*products$. Fig 1 presents the roadmap of the interaction system design with product features.

**Fig 2:**
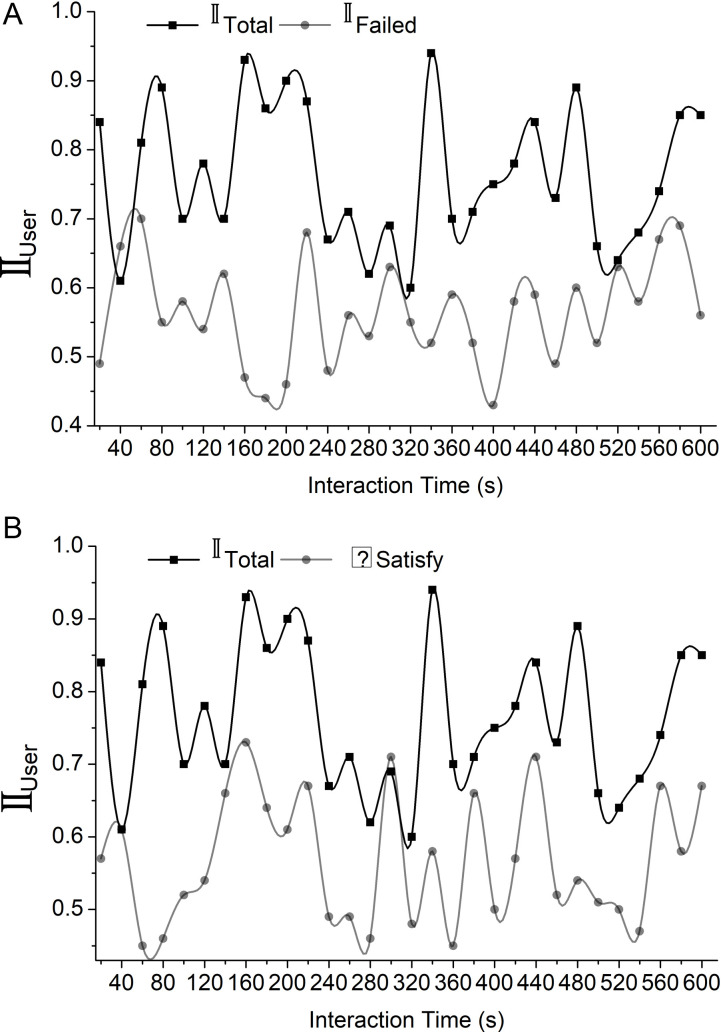
Analysis of $\;{𝕀}_{user}$ **for different interaction times.**

This system is designed with a view of online purchases through e-commerce platforms. From the entire representation, the user experience (in star ratings), reviews, and product reviews are accounted for in this article alone. The dependencies based on quality, feedback, difficulties, user engagement, and interaction rate are extracted and used to analyze the varying order and user reviews. The interaction time variant accounted for is average between the 20s and 600s (https://www.kaggle.com/datasets/datafiniti/consumer-reviews-of-amazon-products). Based on this system, 40 category products are considered with a likelihood where the repetition of product purchase/review is accounted for to update the user experience (Fig 2).

## 3. Interaction-based fuzzy decision support system for improving user experience

The quality of user interaction and design strongly influenced the product’s success in the market nowadays. Integrating decision support systems involves enhancing user experiences by guiding product interactions, leading to the advent of technology. This article introduces an interaction-based fuzzy Decision Support (FDS) system to meet user demands by modifying interaction suggestions. Previous user experiences and reviews to model interaction features were computed based on the FDS system. This enables the identification of fuzzy decisions for interaction improvement or product design modifications. Calculating the probability of various interaction scenarios creates higher-order recommendation variants to enhance user experience. The lower-order variants suggest design improvements, which were analyzed based on the factors. This endeavour delivers superior user experiences and adaptability in interactive platforms making this approach. Interaction platform design feature analysis in products optimizes user experience and needs. Regarding improvements and innovation in the interaction platform, this helps in identifying features. Usability was enhanced by analyzing the interaction patterns. Response availability also plays an important role in developing high-impacting factors. The below equation $\Phi_{analyse}$ computes the analysis of interaction platform factors


$$\Phi_{analyse}=\sqrt{\left({\text{Ƣ}}_{desigh}^{2}+{\text{Ƣ}}_{quality}^{2}+{\text{Ֆ}}_{engage}^{2}+{\text{Ґ}}_{perform}^{2}\right)}\times \left(1+\ln{\mathbb{R}}_{avail}\right)$$ (1)

In the above equation, ${\text{Ƣ}}_{desigh}\;and\;{\text{Ƣ}}_{quality}$ represents the product design and quality of the product. The variable $\;{\text{Ֆ}}_{engage}$ denotes the user engagement and ${\text{Ґ}}_{perform}$ measures the technical performance of the interface, ${\mathbb{R}}_{avail}$ represents the response availability during the interactions between the user and the interface. This also aims to measure the overall analysis of the product design feature’s effectiveness by incorporating various factors that contribute to the product design and user interactions. The interaction between users and an interaction platform determines the overall user experience and satisfaction. Valuable feedback was provided based on user engagement, enhancing the system’s performance. High levels of interaction indicate the engagement of users, which leads to an increased usage rate. The below equation ${𝕀}_{user}$ formulates the interaction of the user with the interaction platform.


$$\;\begin{array}{c} \delta =\frac{{𝕀}_{total}-{𝕀}_{failed}}{{𝕀}_{total}}\\ \exists =\frac{{\text{Ֆ}}_{engage}+{\mathbb{R}}_{avail}+{\text{Ֆ}}_{satisfy}}{\eta }\\ {𝕀}_{user}=\frac{\delta \times \exists }{\sqrt{{𝔇}^{2}+{𝖧}^{2}}}+\Phi_{analyse} \end{array}\}$$ (2)

Here, δ formulates the successful interaction by the user with the interface by incorporating total interaction and failed interaction, which is represented as $\;{𝕀}_{total}\;and\;{𝕀}_{failed}$. Fewer failed interactions than many total interactions ensure an increasing number of successful interactions. The computation of ∃ includes user satisfaction which is denoted as $\;{\text{Ֆ}}_{satisfy}$, user engagement, and response availability. These factors were analyzed based on the system learning rate represented as  η. The overall user interaction with the interaction platform is performed by D, and H represents the interaction difficulty faced by the user and the support system the user needs. It normalizes the interaction quality by incorporating previous computations. This also enhances the interaction quality between the user and the interaction platform. In Fig 2, the analysis of $\;{𝕀}_{user}$ observed under different interaction times is presented. The high and low interaction rate versions heavily impact the design decisions and user experiences. For example, features like real-time feedback or interactive aspects are typically necessary for high interaction rates to keep users engaged. This is typical in contexts where users must remain engaged over an extended period, such as on game platforms. Utility applications, such as schedules or calculators, tend to have low interaction rates since their designers have emphasized making their interfaces as simple as possible to avoid confusing users. To keep the system functional and user-friendly across different use cases, these variances directly influence user satisfaction by adjusting the design to the predicted interaction frequency.

**Fig 3:**
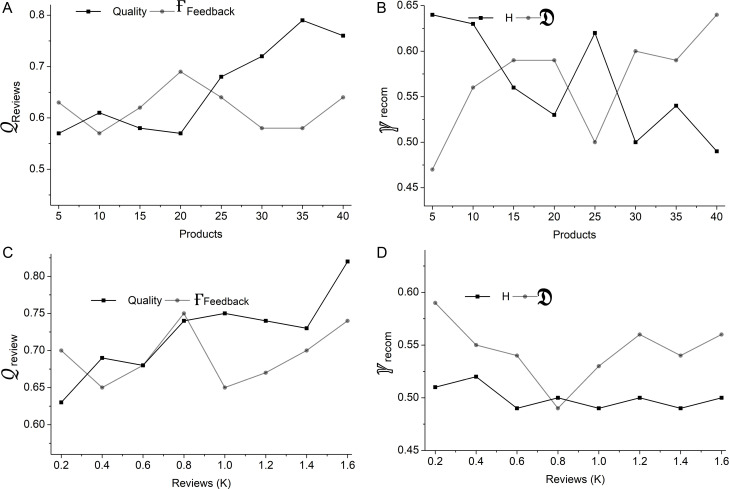
${𝒬}_{review}$ and $\;{\text{γ}}_{reccom}$ for products 5–40.


In this analysis, the failed interaction ${𝕀}_{failed}$ is detected when the number of interacting user ${𝕀}_{user}$ increases with an increase in interaction time. To address the failed interaction, we have to analyze the failed interaction with the total interaction ${𝕀}_{total}$. The successful interaction is computed based on the difference between the total interaction and the failed interaction by the total interaction. The value ranges from 0 to 1, indicating successful over failed interactions. This helps to identify and reduce the failed interaction and aims to improve the platform without failed interactions. This enhances the overall interaction rate, user engagement, and satisfaction (Fig 3). To maintain a good user experience, providing a responsive interaction platform for user inputs is important. The platform enhances user satisfaction by providing an accurate response. A responsive platform can provide valuable insights into user behaviour and improvements. The below equation ${\mathbb{R}}_{interact}$ computes the response given by the interaction platform to the user.

$$\;\begin{array}{c} ∄=\left[\log\left(1+{\text{Ғ}}_{feedback}\right)+{\mathbb{R}}_{avail}+{\text{Ґ}}_{perform}\right]\\ {\mathbb{R}}_{interact}={𝕀}_{user}\times \left[\left[∄-{𝕀}_{total}\right]-D\right] \end{array}\}$$ (3)

The above computation ∄ measures the overall response of the system to the user. This includes factors like ${\text{Ғ}}_{feedback}$ represents the feedback suggestion from the interaction platform to the user, along with the platform’s response availability and technical performance. Thus, the quality of response is evaluated based on this approach by incorporating factors. This provides a response based on the user’s interaction with the platform. Reviews provide feedback collected from users and suggestions from the interaction platform. This helps to identify the areas for improvement. Recommendations obtained from reviews help guide the design process. This ensures that changes regarding user preferences lead to increased satisfaction and engagement and that the product art design meets better user expectations. The below equations ${𝒬}_{review}\;and\;{\text{γ}}_{reccom}$ computes the review and recommendation process.


$${𝒬}_{review}={\text{Ƣ}}_{quality}\times \left[\log\left(1+𝔇\right)+{\mathbb{R}}_{avail}-\left({𝕀}_{total}+{\text{Ғ}}_{feedback}\right)\right]$$ (4)

$${\text{γ}}_{reccom}=\left[\left({\mathbb{R}}_{interact}\times exp\left(𝔇\right)+{𝕀}_{user}\right)-\left({\text{Ֆ}}_{engage}\;+U_{easy}\right)\right]$$ (5)

Here, factors such as product quality, the difficulty faced by the user, and response availability were incorporated to compute the review. This total interaction and feedback suggestion factors were included to enhance the review. The term $\;U_{easy}$ represents the user’s easy accessibility of the interaction platform. This also includes factors like the response generated by the interaction platform, interaction of the user, and user engagement with the platform. Through previous user experiences and reviews, the maximum possible interaction features involve factors incorporating user engagement. The assessments on $\;{𝒬}_{review}$ and $\;{\text{γ}}_{reccom}$ for the different products is given in Fig 3.

**Fig 4:**
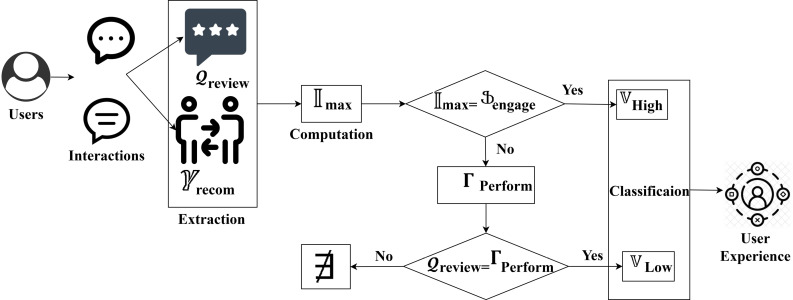
Classification based on interactions.


The ${𝒬}_{review}$ and ${\text{γ}}_{reccom}$ were analyzed based on ${𝕀}_{user}$ which enhances the review and recommendations during the interactions. An increase in ${\text{Ƣ}}_{quality}$ along with the slight increase in ${\text{Ғ}}_{feedback}$ shows how user interaction improves product quality and feedback. The high rise enhances the user’s engagement with the products and reviews on products based on their features. However, some interaction fluctuation may occur due to the difficulties they face. A high recommendation was measured based on the $U_{easy}$ and  𝔇. The recommendation is high when the products meet the expectations, and it is easy to access for the user. There might be a decline due to the difficulties found by the user (Fig 4). The computation of user feedback and reviews provides insights into how users interact with the features. Ultimately, this approach maximizes interaction and overall satisfaction based on the user requirements. The below equation ${𝕀}_{\max}$ computes the maximum possible interaction features based on previous user experiences and reviews.

$${𝕀}_{\max}=\left({\text{Ֆ}}_{engage}\times \log\left({𝕀}_{user}+1\right)\times \left(\frac{{𝒬}_{review}}{{𝕀}_{total}}\right)-\left({\text{Ֆ}}_{satisfy}+{\text{γ}}_{reccom}\right)\right)$$ (6)

This above equation includes factors such as user engagement, user interaction, review, and total interaction by the interaction platform. This also includes user satisfaction and recommendations. The model can predict the design features that enhance user engagement by combining them with historical usage data. This leads to improved user satisfaction and interaction. Based on user interaction experience, it is essential to compute high and low variants in product art design to optimize the design. User engagement and interaction represent high variants. Similarly, user engagement and difficulties that failed to be addressed were computed in low variants. The analyses of both high and low variants obtained a detailed understanding of improvements. This helps to increase positive user experiences and enhance the platform’s interactions.


$$\;\begin{array}{c} {𝕍}_{high}=\left({\text{Ֆ}}_{engage}\times \sqrt{{𝕀}_{user}}\times exp\left({𝒬}_{review}\right)\times \frac{𝔇}{{𝕀}_{total}}\right)+{𝕀}_{\max}\\ {𝕍}_{low}=\left[{\text{Ֆ}}_{engage}\times \left(\frac{\log\left({𝕀}_{user}+1\right)}{1+{𝒬}_{review}}\right)\times \frac{𝔇}{{𝕀}_{total}}\right]-\left({\text{Ґ}}_{perform}+{\text{Ֆ}}_{satisfy}\right) \end{array}\}$$ (7)

**Fig 5:**
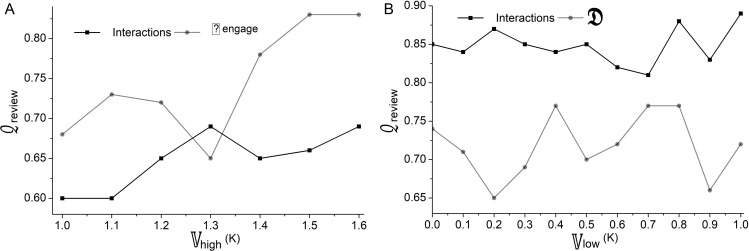
Analysis of interactions, difficulties, and user engagements.

In the above equation, ${𝕍}_{high}\;and\;{𝕍}_{low}$ represents the computation of high and low variants. This helps to improve user satisfaction based on reviews and technical performance. This enhances user interaction in product art design. This also leads to enhanced user satisfaction. This approach ensures the final product effectively meets user needs and preferences. The classification based on interactions as per equation (7) is illustrated in Fig 5.

**Fig 6:**
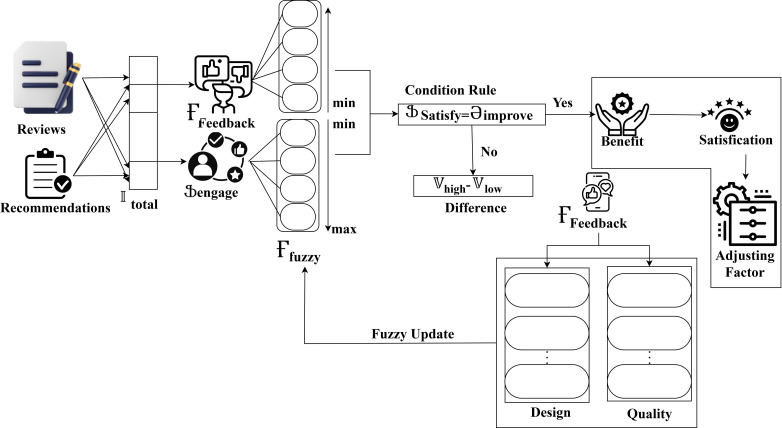
Fuzzy DSS for interactions and preferences.

In Fig 4, the classification process for ${𝒬}_{review}$ and $\gamma_{recom}$ is portrayed. The classification is based on ${𝕀}_{\max}$ and $\Gamma_{perform}$ computations where user satisfaction (on product design) and recommendation (through reviews) are prominent in handling user engagements. If user engagement is the only factor to satisfy the ${𝕀}_{\max}$ then the variant is high for any interaction. This classification on interaction is required to prevent user loss and difficulties faced by the users during product search such that ${𝒬}_{review}$ is the low variant factor. In this classification process, user recommendations under different likelihood factors ease the need for product design improvement. Based on this classification, the review based on interactions, difficulties, and user engagements is analyzed in Fig 6.

In the above analysis, the high and low variants ${𝕍}_{high}$ and ${𝕍}_{low}$ were detected based on the ${𝒬}_{review}$ provided by the user during their interactions. An increase in user interaction and variance in user engagement leads to high variants. An increase in difficulties leads to a decrease in user engagement. Due to high variations in difficulty levels the user faces during their interactions, user engagement also decreases. The user engagement and difficulties that were not addressed led to low variants. This overall analysis of high and low variants helps to improve user satisfaction. This also ensures that the final product meets user needs and preferences (Fig 5).

## 4. Fuzzy decision support system for interaction and preferences

To handle the inherent uncertainty in user interactions and preferences, the principles of fuzzy logic are incorporated and work based on a fuzzy decision support system (FDS). This helps monitor complex human interactions with products where the preferences and experiences of users are not always common. To identify patterns in user behaviour, the collected data from previous user interactions were processed by the FDS system. To quantify these assessments, various design features and interaction methods were evaluated using fuzzy sets and rules. Once the system analyzed the interaction data, the recommendations for enhancing user experience through a two-tiered approach were generated. Higher-order recommendation variants are developed for high interaction success probabilities by suggesting features to maximize user engagement. This consistent and positive feedback received by design elements or interaction techniques was included. Similarly, lower-order variants are incorporated to address areas with potential for improvement. The interaction rate was enhanced by adding new features and modifications. The overall design is based on user feedback, continuously improved by this combined approach, enhancing successful interactions. By continuously refining its recommendations through fuzzy logic, the FDS system aims to provide a more satisfying user experience that adapts to evolving user needs.


$${\text{Ә}}_{benefit}=\min\left(\max\left({\text{Ֆ}}_{engage},\min\left({𝕀}_{user},\exp\left({𝒬}_{review}\right)\right)\right),{\text{γ}}_{reccom}\right)$$ (8)

$${\text{Ә}}_{improve}=\max\left(\min\left(D,\max\left({\text{Ґ}}_{perform}\right)\right),\frac{1}{{\text{Ֆ}}_{satisfy}}\right)$$ (9)

$${\text{Ӻ}}_{fuzzy}=\mu \left[\frac{1}{1+e^{-\left({𝕍}_{high}-{𝕍}_{low}\right)}}\right]\times \left({\text{Ә}}_{benefit}+{\text{Ә}}_{improve}\right)$$ (10)

This ${\text{Ә}}_{benefit}$ computes the system effectively maintained user needs by incorporating these factors. This leads to the enhancement of user engagement. By evaluating these aspects, the interaction platform identified the high interaction features. This is beneficial for the user. The computation ${\text{Ә}}_{improve}$ helps to identify the regions where users struggle to handle the interaction platform. By considering these factors, the platform can understand the user’s difficulties. This ensures the maximum interaction features and helps to identify the improvement needs. The term ${\text{Ӻ}}_{fuzzy}$ helps to accommodate high and low variants. Here, μ represents the membership function. This provides a more comprehensive and balanced analysis. This ensures the overall performance of the interaction platform with the user by incorporating improvement needs and benefit factors. This fuzzy decision system computed the following process of higher-order recommendation and lower-order variants to enhance the specific decision requirements. The fuzzy DSS for the interactions and preferences is illustrated in Fig 6.

**Fig 7:**
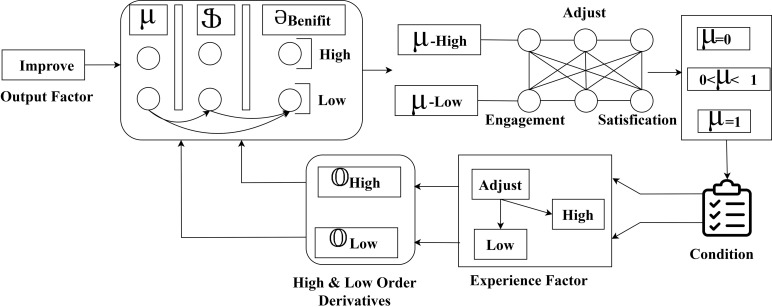
Higher-order derivative-based recommendation process.


The fuzzy derivatives aim to achieve high user satisfaction irrespective of the interaction time and products. This fuzzy process separates ${\text{Ғ}}_{feedback}$ and ${⍰}_{engage}$ from $I_{total}$ from the output identified. The minimum and maximum derivations are identified; the minimum value is 0 for the feedback and >0 for engagement. Therefore, the derivatives need to satisfy $\partial_{improve}$ condition rule. If this rule is satisfied, an adjustment factor will be derived for multiple benefits. In the case of rule base difference between ${𝕍}_{high}$ and ${𝕍}_{low}$ is estimated for ${\text{Ғ}}_{feedback}$. Therefore, the design and quality derivatives are utilized and extracted to maximize the fuzzy process. This fuzzy process ensures high ${⍰}_{satisfy}$ for different products (Fig 7). In developing multiple higher-order recommendation variants, the fuzzy process involves using fuzzy logic to handle the variability in user satisfaction and feedback. Various user data and feedback were computed and processed through fuzzy inference systems in this process. By combining higher-order recommendations, user satisfaction is enhanced to improve product quality. The fuzzy system uses a membership function to handle inputs. This allows for a range of recommendation options for different user needs. This approach also enhances the user experience by improving overall interaction. Including multiple factors ensures that recommendations are modified to evolve user expectations and feedback. The below equation ${𝕆}_{High}$ computes the derivation of higher-order based on a fuzzy decision system.

$$\;\begin{array}{c} \mu_{factor-High}=\left({\text{γ}}_{reccom}+{𝒬}_{review}+{𝕀}_{user}+{\text{Ֆ}}_{satisfy}\right)\\ \mu_{adjust-High}=\left[\mu_{factor-High}\times {\text{Ғ}}_{feedback}\times {\text{Ә}}_{improve}\right]\\ {𝕆}_{High}=\log\left(1+\mu_{adjust-High}\right) \end{array}\}$$ (11)

$${{\text{Ƣ}}_{desigh}}_{imp}=\left[\log\left({\text{Ғ}}_{feedback}+1\right)\times exp\left({𝒬}_{review}\right)\right]\times \left[{\text{Ƣ}}_{quality}-\left(\sqrt{𝔇}+{𝕆}_{High}\right)\right]$$ (12)

**Fig 8:**
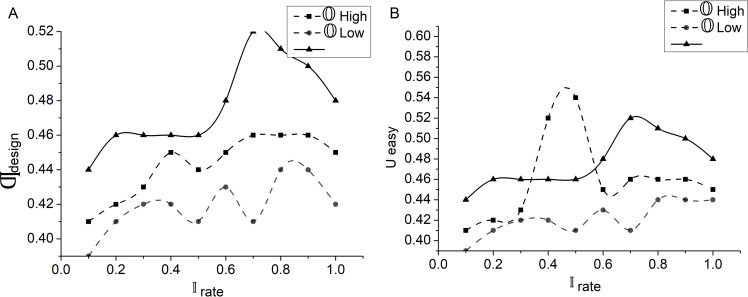
Analysis of interaction rate for design modification and user adaptability with adjustments.

From the above equation, the computation of ${{\text{Ƣ}}_{desigh}}_{imp}$ represents the improvement in the product design based on the higher-order derivation. The term $\log\left({\text{Ғ}}_{feedback}+1\right)$ represents that an increase in user feedback leads to the improvement of the product. The term $\exp\left({𝒬}_{review}\right)$ measures the importance of the review, which plays a major role in product improvement. Incorporating product quality ensures that the higher quality leads to the product improvement. The difficulty part denotes the problems that are noticed concerning improvement. In Fig 8, the higher-order derivative-based recommendation process is illustrated.

The high and low-order derivatives are identified from $\partial_{improve}$ output factor. First μ to ⫏∣ and μ to $\partial_{benifit}$ mapping to differentiate μ−High and μ−Low is performed. These variants ⫏∣ represent the low and $\partial_{benifit}$ represent the high output. The user engagement and satisfaction are classified as =0, 0<μ<1, and μ=1 variants. The conditions inferred in Fig 7 are referenced to estimate the experience factor through different adjustment factors. Therefore, if the adjustment is based on high μ, then ${𝕆}_{High}$ is the variant, and the rest is ${𝕆}_{Low}$. Both these derivatives are updated for the μ and $\partial_{benifit}$ to maximize suggestions (Refer to Fig 8), it is essential to compute lower-order derivation to increase interaction rates to recommend different design features. These include factors like product design, user interaction, and user engagements that are adjusted based on user feedback in the interaction platform. The impact on user engagement was derived from the feedback. This enhances the process and leads to modifications in the interaction rate. The modification process is easier for lower-order derivatives than for higher-order derivatives. This evaluates the performance and features that most effectively enhance user interaction. This ensures enhancement in user experiences and interaction patterns. Ultimately, this method leads to the improvement and enhancement in overall user interaction rates. The below equation ${𝕆}_{Low}$ computes the lower-order derivatives of the user with the interaction platform to improve the interaction rate.


$$\;\begin{array}{c} \mu_{factor-Low}=\left({\text{Ƣ}}_{desigh}+{𝕀}_{user}+{\text{Ֆ}}_{engage}\right)\\ \mu_{adjust-Low}=\mu_{factor-Low}\times \left[\left(1-{\text{Ғ}}_{feedback}\right)\times \left(1-{\text{Ә}}_{benefit}\right)\right]\\ {𝕆}_{Low}=\left[\exp\left(\mu_{adjust-Low}\right)-1\right]+U_{easy} \end{array}\}$$ (13)

$${{𝕀}_{rate}}_{imp}=\left[\log\left(1+{\text{Ֆ}}_{engage}\right)\times \log\left(1+U_{easy}\right)\right]-\left({𝕆}_{Low}+D\right)$$ (14)

Here, ${{𝕀}_{rate}}_{imp}$ represents the improvement in interaction rate based on the low-order derivations. The term $\log\left(1+{\text{Ֆ}}_{engage}\right)$ measures how user engagement contributes to the increased interaction rate. Then the term $\log\left(1+U_{easy}\right)$ measures the easy usage of interaction platforms, which leads to improved interaction rates. Difficulties handling the platform are to be addressed to enhance the interaction rate. An increase in interaction rate enhances the overall performance of the interaction platform and user. The analysis of the interaction rate for different design modifications and user adaptability is presented in Fig 8. This analysis follows the low and high adjustment factors along the illustrations.

The analysis of ${{\text{Ƣ}}_{desigh}}_{imp}$ carried with ${𝕆}_{High}$ higher order derivation. The improvement in the product design is based on the 𝔇 and ${\text{Ғ}}_{feedback}$ obtained from the users. An increase in difficulties shows the difficulties faced by the user during the handling of the product or interaction. An increase in feedback measures the user’s needs and ideas towards the product’s design and features. This high amount of feedback and difficulties has led to product design improvement. The analysis of ${{𝕀}_{rate}}_{imp}$ is carried along with lower-order derivation ${𝕆}_{Low}$. The factors obtain the interaction rate improvement $U_{easy}$ and ${\text{Ֆ}}_{engage}$ based on the user’s interaction with the products. An increasing rate of easy usage of products leads to an increasing user engagement rate with the products. This automatically leads to an improvement in the interaction rate. An increase in interaction rate enhances the overall performance of the interaction platform and user. This enhances user satisfaction and engagement based on the interaction with the product (Fig 8).

## 5. Data-based analysis

The data sources used earlier are inherited to perform the analysis based on several products and likelihood variants. These variants analyze user adaptability, experience assessment, interaction rate/ product, design modification, and recommendation ratio. The interaction time factor for both variants varies between 50s and 600s.

### Influence of number of products

**Fig 9:**
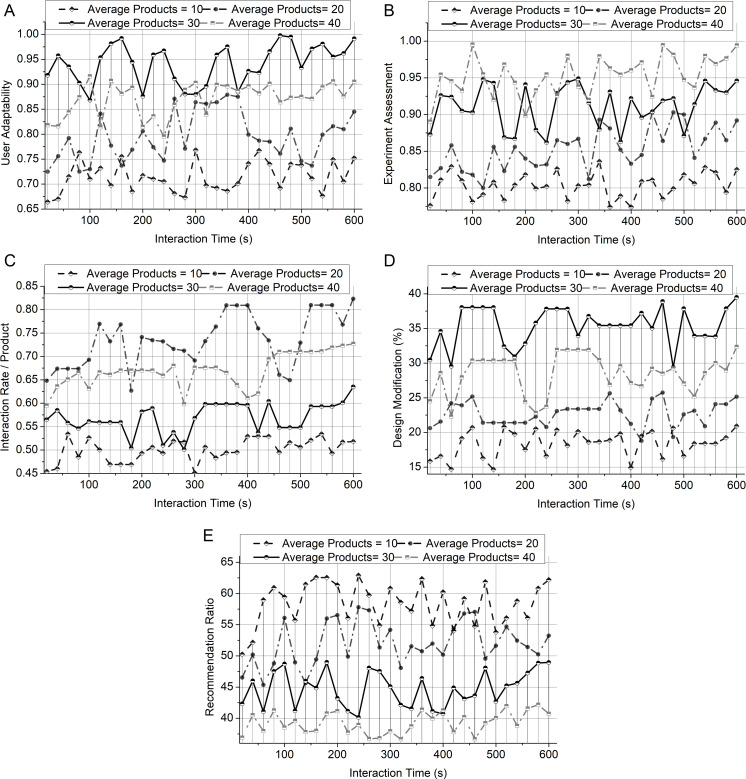
Influence of interaction time and avg. Products on different parameters. Influence of likelihood. (a) User adaptability. (b) Experience assessment. (c) Interaction rate/product. (d) Design modification. (e) Recommendation ratio.

The number of products enhances the computation factors based on user engagement and satisfaction. When the number of products is 10, it indicates a high recommendation ratio based on the user’s engagement with the product. When the number of products is 20, it shows a strong engagement among users and increases the high interaction rate for the product. When the number of products is 30, suggest the high user adaptability and design modifications based on user feedback and interaction. When the number of products is 40, it indicates an increase in experience assessment among overall user engagement. The cumulative representation of the number of product influences is portrayed in Fig 9.

**Fig 10:**
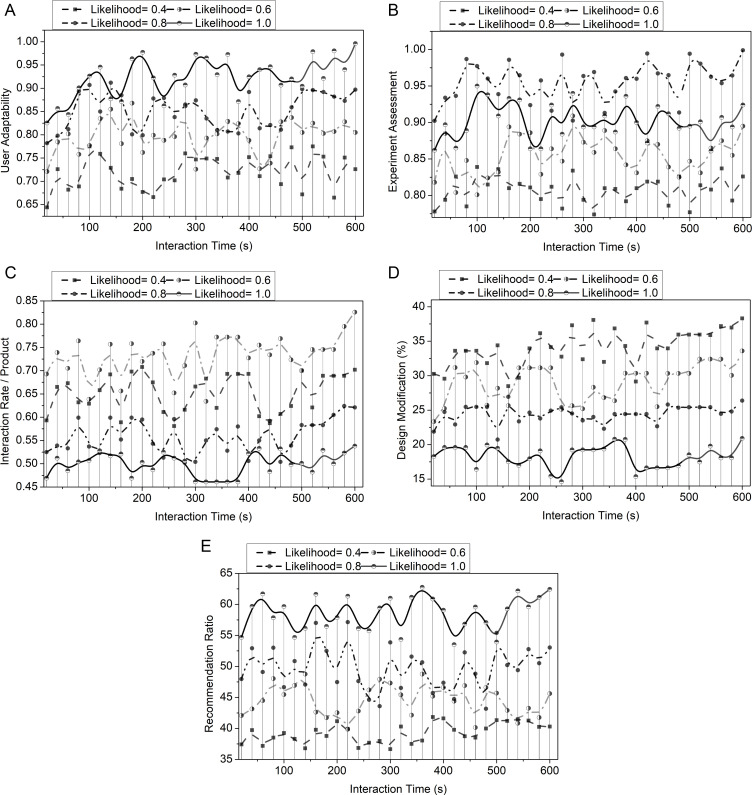
Influence of interaction time and likelihood on different parameters. (a) User adaptability. (b) Experience assessment. (c) Interaction rate/product. (d) Design modification. (e) Recommendation ratio.

Fig 10 illustrates the influence of likelihood and interaction time over different parameters. The Likelihood variant is computed based on the product’s interaction with users based on its features and designs. This measures how the likelihood values impact the user adaptability, experience assessment, design modification, interaction rate, and recommendation ratio. The low likelihood indicates an increase in user adaptability and recommendation ratio. Positive feedback from users measures an increase in experience assessment in the likelihood. This high likelihood shows that the products meet users’ needs and satisfaction. The moderate likelihood shows a high interaction rate, resulting in high user engagement with the products. When the likelihood value is between low and moderate, the user suggests a high modification for the design.

## 6. Discussion

User adaptability is calculated in relationship with interaction time for better results. Fluctuations in adaptability indicate that there is no linear interaction over time. Initially, an increase in user adaptability shows that the product interface becomes more familiar to users. The interaction between the user and the interaction platform is computed as ${𝕀}_{user}\;and\;{\mathbb{R}}_{interact}$ which results in enhancing user adaptability. This helps to maintain a positive learning rate and reliable outcome. Adaptability is decreased when users face challenges or difficulties in handling the platform. The successful platform handling by the user increases interaction time and adaptability. The adaptability increases values as the interaction time increases towards the upper limit. This suggests that high user satisfaction or engagement is based on the high interaction with the product [Figs 9(a), 10(a)].

During the analysis of experience assessment, the fluctuation in the interaction time suggests that the experience faced by the overall users. The experience assessment is calculated based on the computation of ${𝒬}_{review}\;and\;{\text{γ}}_{reccom}$ represents the review and recommendations. This enhances user engagement based on the previous recommendations and reviews provided by the interaction platform. Initially, the increase in experience assessment shows users’ engagement with the products and reviews on products based on their features. However, some interaction fluctuation may occur due to the difficulties faced by users or the platform’s technical performance. During the end of the interaction time, the assessment shows higher values, which results in more interaction and positive experience outcomes. Continuously maintaining a high user experience in the interaction period ensures a positive impression among users [Figs 9(b), 10(b)]. When users explore more features and products, the interaction rate will increase and show a peak during some time intervals. The maximum possible interaction features based on previous user experiences and reviews are computed as $\;{𝕀}_{\max}$. The high interaction rate ${{𝕀}_{rate}}_{imp}$ is computed based on the low-order fuzzy derivative $\;{𝕆}_{Low}$. This constant peak in intervals indicates the increasing rate of user interaction by particularly focusing on some products and their quality. This suggests user engagement and satisfaction stability, enhancing the interaction rate. When the product quality and features meet the user’s needs, the interaction rate will not decline. This shows that the product’s design, quality, and features should be made according to the user’s need to engage users with the interaction platform continuously. This leads to a consistent increase in interaction rate and user engagement [Figs 9(c), 10(c)]. The design modification is based on the user’s reviews and the interaction time. The fluctuation during the interaction time indicates the varying levels of suggestion that occur for the modification of designs. The user provided feedback on design modifications of product features and designs. From the proposed work, the computation of feedback is represented as $\;{\text{Ғ}}_{feedback}$. Based on the user’s feedback, the design should be iteratively improved, and the modification process should be stabilized. The improvement in design is computed as ${{\text{Ƣ}}_{desigh}}_{imp}$ with the help of ${𝕆}_{High}$ represents higher-order fuzzy derivatives. By combining higher-order recommendations, user satisfaction is enhanced to improve product quality. This improves the user engagement with the products. This helps to improve the product design, where continuous feedback and interaction rates enhance user satisfaction [Figs 9(d), 10(d)]. This recommendation ratio measures the likelihood of user recommendations based on the product design and quality. Recommendations obtained from reviews help guide the design process. This ensures the changes regarding user preferences. In the proposed work, the computation of ${\text{γ}}_{reccom}$ measures the recommendation. This is formulated along with factors like interaction rate, difficulties faced by the users, ease of accessing the interaction platform, and user engagement. The recommendation ratio is initially high when the products meet the users’ expectations. This indicates an increase in user satisfaction. To recommend different design features, it is essential to compute lower-order derivation to increase interaction rates. The fluctuation in the interaction period shows the users’ positive and negative feedback of the product. When the products are based on the user’s need, it may increase the recommendation ratio and user engagement toward them [Figs 9(e), 10(e)].

Using membership functions and fuzzy logic principles, the system converts user inputs that are either incompatible or imprecise into degrees of confidence. When consumers say comments like “somewhat comfortable” or “a bit unclear,” the algorithm takes these as half-truths instead of lying down the road. It considers this information when analyzing additional data points to spot patterns or preferences. Iterative feedback loops are also a part of the IB-FDSS, which allows it to improve its suggestions in response to more precise or clear input, guaranteeing trustworthy results even when there is initial uncertainty.

## 7. Summary

User interaction experiences were enhanced significantly in product art design by integrating a fuzzy Decision Support (FDS) system. The FDS system effectively models the interaction in features and identifies necessary improvements or design modifications using user feedback from past interactions. The use of higher-order recommendation variants boosted user engagement. In contrast, lower-order variants guided the product design enhancements. The interactive platforms improve the overall quality of user experience, which can continuously evolve to meet user needs. This ensures the adaptiveness of the decision-making process. To highlight its potential to change product design and user interaction, fuzzy logic in decision support demonstrates a robust methodology for the proposed system. This methodology aimed to improve the interaction rate of 97.4% with better impacts on product design and modification using likelihood variants. The user experience assessment is performed using the higher-order variants with a better user adaptability rate of 98.9%, maximizing the recommendations.
